# Profile of C-reactive protein, white cells and neutrophil populations in febrile children from rural north-eastern Tanzania

**DOI:** 10.11604/pamj.2017.26.51.10264

**Published:** 2017-01-31

**Authors:** Coline Mahende, Billy Ngasala, John Lusingu, Thomas Mårtensson, Paminus Lushino, Martha Lemnge, Bruno Mmbando, Zul Premji

**Affiliations:** 1 Korogwe Research Station, Tanga Centre, National Institute for Medical Research, P. O. Box 5004, Tanga, Tanzania; 2 Department of Medical Entomology and Parasitology, School of Public Health, Muhimbili University of Health and Allied Sciences, P. O. Box 65001, Dar es Salaam, Tanzania; 3 Department of International Health, Microbiology and Immunology, University of Copenhagen, Denmark; 4 Department of Clinical Science, Intervention and Technology (CLINTEC), Karolinska Institutet, Stockholm, Sweden

**Keywords:** C-reactive protein, children, Tanzania

## Abstract

**Introduction:**

C-reactive protein (CRP), white blood cell (WBC) and absolute neutrophil counts (ANC) are important inflammatory biomarkers in the early diagnosis of infections. However, little is known on their profile and usefulness in fever case management in children attending outpatient clinic in rural north-eastern Tanzania.

**Methods:**

Patients aged between 2 and 59 months presenting with fever at Korogwe District Hospital were enrolled. Venous blood was collected for evaluation of serum CRP, WBC and ANC. Individual patient diagnosis was based on integrated management of childhood illness (IMCI) guidelines and laboratory investigations (blood and urine cultures).

**Results:**

A total of 867 patients were enrolled, out of which 691 (79.7%) had complete clinical and laboratory data available for analysis. Acute upper respiratory tract infection 284 (41.1%), acute gastroenteritis 127 (18.4%) and pneumonia 100 (14.5%) were the most frequent diagnoses. The geometric mean levels of serum CRP, WBC and ANC were 10.4 (95% CI: 9.2 - 11.8), 11.5 (95% CI: 11.1 - 11.9) and 5.5 (95% CI: 5.2 - 5.8), respectively. CRP≤20, WBC≤15 (10^3^cells/µL) and ANC≤10 10^3^cells/µL) were observed in the majority of the patients with upper respiratory tract infection, pneumonia, acute gastroenteritis and non-specific febrile illness. Only serum CRP levels were positively correlated with positive blood cultures at a calculated cut-off value of 37.3 mg/L, giving a specificity of 77.8% and sensitivity of 74.2%.

**Conclusion:**

CRP assessment together with IMCI guidelines may be useful in assisting the diagnosis and management of paediatric febrile infections in Tanzania.

## Introduction

C-reactive protein (CRP), white blood cell (WBC) and absolute neutrophil counts (ANC) are important inflammatory markers used in the early detection of acute infections in children and neonates [1, 2]. These tests are routine analyses in developed countries used to guide the clinicians in discriminating viral from bacterial infections as well as to monitor the response to antibiotics [3, 4]. Little has been published on their utility in aiding diagnosis of infections among patients presenting with acute febrile illness at outpatient clinics in rural Tanzania [5].

Febrile infectious childhood illnesses represent a major global health problem. In Tanzania where malaria cases are declining, acute respiratory tract infections of viral origin have been reported to be the leading cause of such illnesses [6, 7]. Frequently, these infections are presumptively treated with antimicrobials due to lack of definite diagnostic tools in healthcare facilities [8, 9]. Tanzania is among the countries that use the Integrated Management of Childhood Illness (IMCI) guidelines in classifying the diagnosis and management of most childhood diseases [10]. IMCI promotes rational use of antimalarial drugs but has remained unspecific in the use of antimicrobials [11]. The cause of infection (whether bacterial or viral) can not be differentiated by IMCI guidelines alone unless complete laboratory investigations are performed. Blood culturing and molecular analyses for identification of bacterial and viral organisms are usually unavailable and time consuming [12, 13]. Thus there is a need for inflammatory markers that can assist in the early detection of infections for guiding optimal use of antimicrobials. These markers will have to be rapid and cost effective for use in low and middle-income countries [14].

CRP has been shown to be a rapid useful predictor of bacterial infection and has guided clinicians in reducing antimicrobial use [15, 16]. However, there are considerably varying data regarding the sensitivity and specificity of CRP as a marker in predicting bacterial infections [17]. Despite these reports, understanding serum levels of CRP in febrile children presenting at different health care settings is crucial towards improved rational use of antimicrobial drugs. WBC and ANC have been used alongside clinical symptoms to predict severe bacterial infection and to discriminate between viral and bacterial causes of pneumonia in children [18, 19]. ANC had been reported to be a better diagnostic indicator than WBC for bacterial infections [20]. However, like CRP, the sensitivity and specificity performance of both WBC and ANC have been reported to vary considerably [21, 22]. Furthermore, it is important to understand their performance in different health care contexts, particularly in sub-Saharan Africa where few studies of the usefulness of these markers in addition to IMCI for improved management of childhood fevers have been conducted. The aim of the study was to evaluate the profile and utility of CRP levels, WBC and ANC in aiding clinical diagnosis among children presenting with fever at outpatient clinic in rural north-eastern Tanzania.

## Methods

### Study area and population

The study was conducted between January and October 2013 at Korogwe District Hospital (KDH) in north-eastern Tanzania. Approximately 73,275 children under the age of five years live in Korogwe District [23]. The hospital receives around 6000 (2012 estimates) outpatient visits from children under-five annually. The administrative coverage for routine childhood vaccines has been above 90% (Korogwe Primary Health Care report, District Medical Officer, personal communication). These vaccines include; BacilleCalmette–Guérin, pentavalent vaccine (diphtheria–tetanus–pertussis, hepatitis B and haemophilus influenza type B), polio and measles. Pneumococcal and rotavirus vaccines were introduced in Tanzania by the Immunizationa and Vaccines Development Department (IVD) as part of the childhood routine immunization in January 2013.

### Enrolment of study participants

Sick children presenting at KDH outpatient clinic were screened for study eligibility. The inclusion criteria were: children aged between 2 and 59 months presenting at KDH with a history of fever in the last 48 hours or measured axillary temperature >37.5°C at presentation. Exclusion criteria included previous consultation for the presenting complaints, intake of antimicrobial and/or antimalarial drugs within the last seven days, planned admissions (e.g. elective surgery) and trauma/injury.

### Ethics statement

The study was granted ethical clearance with reference number NIMR/HQ/R.8a/Vol.1X/1373 from the Tanzanian Medical Research Coordinating Committee. Parent/legal guardian of every child enrolled in the study provided a written informed consent by either signature or thumb print for the illiterate parent/legal guardian.

### Study procedure and laboratory analysis

Medical history and clinical examination based in IMCI were performed on each patient and information was entered into a standardised case record form. This included demographic information, clinical history, vital signs, body weight, signs of dehydration, neurological and physical examinations (skin, abdominal, ear, mouth and throat). A chest radiograph was requested if pneumonia was suspected. The final individual patient diagnosis was based on presenting signs and symptoms according to IMCI guidelines and results from available laboratory investigations [10, 24].

Blood for identification of *Plasmodium falciparum*, human immunodeficiency virus, bacteraemia and urine cultures were collected and analysed as previously described in detail [25]. Serum for CRP analysis was obtained after centrifugation (at 3000 rpm for 10 minutes) of 1 ml venous blood collected in plain vacutainer dry tubes. CRP levels were measured using Cobas c111 biochemistry analyser (Roche diagnostics, Indianapolis, IN). Approximately one ml of venous blood was collected in anticoagulated ethylene diamine tetra acetic acid (EDTA) tubes for the analysis of WBC and ANC using an automated 5 –part differential MS4s haematology analyser (Melet Schloesing Diamond Diagnostics, USA). Patient samples were analysed within 4 hours after collection and in-case of delay samples were stored at 2°C - 8°C or -20°C for later analysis.

### Quality control and assessment

Laboratory investigations were carried out at Korogwe Research Laboratory of the National Institute for Medical Research, Tanzania. Quality control was performed daily for each parameter before analysis of patient samples. All procedures were performed according to standard guidelines and instructions from instrument manufacturers [26].

### Power consideration, data management, statistical analysis and definitions

Data were double entered and validated using Microsoft Access database. Data analysis was performed using R version 3.2.3 and STATA version 11.2 (Stata Corp LP, College Station, Texas, USA). Variables were summarized as frequencies and percentages, means, medians, standard deviations and inter-quartile ranges as appropriate. Continuous data that were not normally distributed were transformed using log and geometric means with 95% confidence intervals were reported. Receiver operating characteristic (ROC) curves analysis was performed in R, where sensitivity, specificity and areas under the curve were obtained for different levels of CRP, WBC and ANC using bloodstream bacterial culture results as the gold standard.

This study utilised data collected to estimate the prevalence of different infections among outpatient febrile children. The prevalence of bacterial infection from the study area was assumed to be 13%. The sample size of 861 was estimated to attain a power of 80% at 5% level of significance, with a significance margin (width of confidence interval) of 5% and attrition rate of 15%. Out of these individuals, 660 had complete data for CRP and 643 for WBC which were compared to the bloodstream bacterial culture as a gold standard. This sample size was sufficient to provide the power of 78% for CRP and 79.3% for WBC, with sensitivity of 77.8% and 58% with a precision of 10% and 5% levels level of significance, respectively.

CRP threshold values of ≤ 20 mg/L and > 40 mg/L were used to rule out and rule in probable bacterial cause of infection respectively [27]. WBC and ANC threshold values of > 15x103cells/µl and > 10 ×103cells/µl respectively, were used to classify presence of infection respectively [27].

## Results

### Demographic and clinical characteristics

A total of 867 patients with fever were enrolled in the study. Seventy four (8.5%), 26 (3.0%) and 76 (8.8%) patients did not have samples for laboratory analysis of CRP, WBC and ANC respectively. The remaining 691 (79.7%) patients had complete clinical and laboratory data available for analysis, while 660 (95.6%) had also blood culture results. The median age was 15 months (interquartile range (IQR): 8.5 – 29.2). Girls accounted for 328 (47.5%) of patients. The majority of patients had acute upper respiratory tract infection 284 (41.1%), acute gastroenteritis 127 (18.4%) and pneumonia 100 (14.5%). Other clinical diagnoses are indicated in Table 1. Out of 691 blood cultures collected, only 17 (2.5%) patients had confirmed bloodstream bacterial infection and the leading organism was Salmonella typhi 13 (76.5%). A total of 290 (41.9%) urine samples were cultured and 50 (17.2%) had growth of clinically significant organism with Escherichia coli 28 (56%) as the predominant organism.

**Table 1:** Demographic and clinical characteristics of the 691 analysed patients

Characteristic	Number (%)
**Demographic**	
**Gender**	
Girls	328 (47.5)
Boys	363 (52.5)
**Age categories**	
2 - 11 months	287 (41.5)
12 - 35 months	300 (43.4)
36 - 59 months	104 (15.1)
**Presenting symptoms**	
Axillary temperature °C (IQR)	38 (37.5 - 38.6)
**Duration of fever**	
≤ 24 hours	256 (37.0)
> 24 - ≤ 48hours	435 (62.9)
**Final diagnosis**	
Acute upper respiratory tract infection	284(41.1)
Pneumonia	100 (14.5)
Acute gastroenteritis	127 (18.4)
Malaria	56 (8.1)
Typhoid fever	13 (1.9)
Urinary tract infection	10 (1.4)
Other infections	43 (6.2)
Non-specific febrile illness	58 (8.4)

### Serum CRP levels, WBC and ANC

The geometric mean levels of serum CRP, WBC and ANC were 10.4 (95% CI: 9.2 - 11.8), 11.5 (95% CI: 11.1 - 11.9) and 5.5 (95% CI: 5.2 - 5.8), respectively. The distribution of CRP levels, WBC and ANC as per threshold values according to individual patient diagnosis are shown in Table 2. Over half of patients with upper respiratory tract infection, pneumonia, acute gastroenteritis and non-specific febrile illness had levels of CRP≤20, WBC≤15 (10^3^cells/µL) and ANC≤10 10^3^cells/µL). Patients with malaria 45/56 (80.4%), typhoid fever 12/13 (92.3%) and urinary tract infection 25/50 (50%) were observed to have elevated CRP levels of > 40mg/L but with WBC≤15 10^3^cells/µL) and ANC≤10 10^3^cells/µL).

**Table 2:** Distribution of CRP levels, WBC and ANC according to final diagnosis

Final patient diagnosis	Total	CRP (%)		WBC (%)		ANC (%)	
		≤ 20 mg/L	> 40 mg/L	≤ 15 (10^3^cells/µl)	> 15 (10^3^cells/µl)	≤ 10 (10^3^cells/µl)	> 10 (10^3^cells/µl)
URTI	284	173 (60.9)	58 (20.4)	206 (72.5)	78 (27.5)	236 (83.1)	48 (19.9)
Pneumonia	100	55 (55.0)	24 (24.0)	69 (69.0)	31 (31.0)	81 (81.0)	19 (19.0)
Acute gastroenteritis	127	84 (66.1)	24 (18.9)	94 (74.0)	33 (26.0)	101 (79.5)	26 (20.5)
Malaria	56	2 (3.6)	45 (80.4)	50 (89.3)	6 (10.7)	53 (94.6)	3 (5.4)
Typhoid fever	13	0	12 (92.3)	12 (92.3)	1 (7.7)	12 (92.3)	1 (7.7)
UTI	10	2 (20.0)	5 (50.0)	6 (60.0)	4 (40.0)	8 (80.0)	2 (20.0)
Other infections^a^	43	18 (41.9)	13 (30.2)	30 (69.8)	13 (30.2)	33 (76.7)	10 (23.2)
NSFI	58	31 (53.4)	19 (32.8)	43 (74.1)	15 (25.9)	49 (84.5)	9 (15.5)

URTI, Upper respiratory tract infection; UTI, Urinary tract infection; NSFI, Non-specific febrile illness;

a Dysentery (2), Gingivitis (2), Otitis media (4), Conjuctivitis (4), Human immunodeficiency virus (5), Candidiasis (7), Asthma (8), skin infection (11).

**Figure 1:**
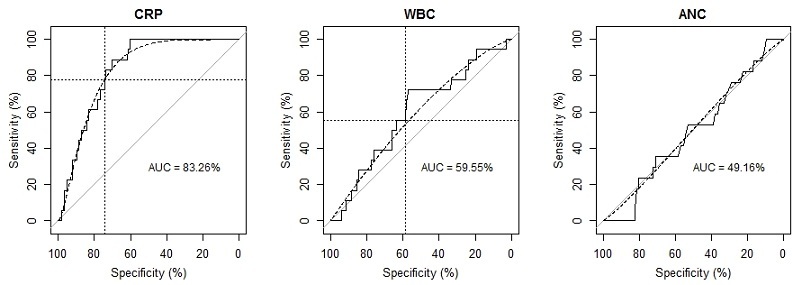
Receiver operating characteristic (ROC) curve (solid line) superimposed with a smooth line (dotted line) for CRP, WBC and ANC respectively. The dotted lines (horizontal and vertical) show the point estimates where specificity and sensitivity lines cross

The ROC analysis was performed taking results from bloodstream bacterial culture as the gold standard (Figure 1). Results show that CRP levels were positively correlated with positive cultures where increase in CRP levels was associated with increased probability of bacterial infection in the blood. The area under the curve (AUC) for this analysis was 83.3%. The optimal cut-off value of CRP for diagnosis of bacterial infection was calculated to 37.3 mg/L, giving a specificity of 77.8% and sensitivity of 74.2% compared with gold standard. Using this CRP cut-off value (37.3 mg/L), 74.2% of individuals were correctly diagnosed. There was weak association between the WBC levels and positive blood cultures. The AUC for WBC was 59.6% (95% CI: 46.6 - 72.5), which is just 9.6% points above the non-informative diagnostic test with AUC of 50%, Figure 1. The results demonstrated that the optimal cut-off value for WBC for diagnosis of bacterial infection was 12.94 x 10^3^cells/µl giving specificity of 58.4% and sensitivity of 55.5%. There was no association between the ANC and bacterial infections in the blood, as seen in Figure 1, the AUC was 49.2%.

## Discussion

The present study has described the profile of serum CRP, WBC and ANC levels among patients under five years of age presenting with fever at outpatient clinic in rural north-eastern Tanzania. Our results indicate that CRP is a better biomarker than WBC and ANC in guiding diagnosis of infections among children with acute febrile illness, especially in infections of bacterial origin. The CRP cut-off values of < 20 mg/L and > 40 mg/L had been demonstrated in ruling out and ruling in bacterial infection respectively [28, 29]. Using these threshold values, half of patients in the study were observed to have CRP levels of < 20 mg/L. The majority of these patients had clinical diagnosis of acute upper respiratory tract infection, pneumonia and acute gastroenteritis. This indicates absence of bacterial cause similar to published data from previous studies [7, 30, 31]. In these cases, clinicians should consider not to treat the child with antimicrobial drugs. CRP testing had been reported by a number of researchers to be significantly helpful in reducing the prescription of antimicrobial drugs especially among patients with respiratory tract infections [32–34].

Our results show that a CRP cut-off threshold value of 37.3 mg/L (approximately 40 mg/L) identified three-quarters of patients with culture proven bloodstream bacterial infection. This observation concurs with data published by Carrol and colleagues in which CRP levels were significantly high among Malawian children with invasive bacterial infection [35]. Patients with malaria infection were also shown to have elevated levels of CRP. The limited evidence from previous research studies conducted in Angola and Tanzania that investigated CRP levels among malaria patients also documented similar findings [36, 37].

Among patients with confirmed bloodstream and urinary tract infections, both WBC and ANC demonstrated poor performance as diagnostic indicators of bacterial infection. This finding is similar to that documented by other researchers in which both WBC and ANC were less reliable indicators in assisting diagnosis of bacterial infections [21, 27, 38]. Furthermore, the observed performance of ANC is in contrast to that published by Christopher and colleagues. Their data indicated high neutrophil count to be one of predictors for bacterial infection among children under five years of age from Tanzania [39]. These varying reports highlight uncertainty of using neutrophil counts in predicting infections of bacterial origin in febrile children.

Currently, Tanzania relies on the use of IMCI guidelines on the diagnosis of febrile illnesses among paediatrics [40]. These guidelines are known to be non-specific as they are based on clinical assessments of fever cases and therefore unable to identify causative agents (bacteria, virus) of infections. CRP marker could be a useful predictive tool in assisting diagnosis of probable bacterial aetiology causing febrile infections. Analysis using CRP rapid diagnostic test have shown to be cost effective and more easily accessible than blood culturing for prompt and appropriate antimicrobial treatment among paediatrics in countries with limited microbiology facilities [41, 42]. Over half of patients in the study had fever lasting 24 to 48 hours. CRP levels peak at 24 to 48 hours after onset of an infection [43], which coincide with the care seeking pattern in rural settings of Tanzania where a majority of patients delay to seek hospital care [44–46]. CRP testing combined with IMCI guidelines could assist diagnosis of causative agents of febrile infections in reducing the unnecessary prescription of antimicrobials. This is important especially in the era of increasing global antimicrobial resistance, epidemiological change of infections (malaria, bacteraemia) and introduction of pneumococcal vaccine.

**Limitation:** Identifying patients who had prior use of antimicrobials was based on information provided by parents and legal guardians. The cut-off levels for CRP were determined from studies conducted in high-income countries that tend to have different ethnicity and pattern of infections [47, 48]. The cut-off levels for CRP can create diagnostic uncertainty when values fall between upper and lower cut-off levels. Sensitivity of blood culture is known to be as low as 40% due to difficult sampling and prior use of antimicrobial drugs, hence fewer positive cultures were observed [49, 50]. Evidence based analysis of viruses causing febrile illnesses such as acute respiratory infection and gastroenteritis could not be performed. Investigations of these viruses could confirm causative agent of such infections for patients with low and normal serum levels of CRP. The sample size (867 patients) for this study was estimated to investigate common causes of acute fever in outpatient children from Korogwe District hospital. This sample size was affected by low sensitivity of blood cultures as well as for CRP, WBC and ANC analyses. Together with unavailability of data from 176/867 (20.3%) decreased substantially the power and is likely to have biased findings of this study.

## Conclusion

The results indicate that CRP test may be useful in assisting the diagnosis of febrile infections in children. The use of CRP test together with IMCI guidelines may improve rational use of antimicrobial drugs among febrile paediatrics in Tanzania.

### What is known about this topic

- CRP test is a routine analysis for early detection of acute infections in children and neonates in developed countries;
- CRP has been shown to be a rapid useful predictor of bacterial infection and has guided clinicians (from developed countries) in reducing antimicrobial use.

### What this study adds

- Evidence based data of CRP levels of febrile children from rural Tanzanian. The data indicate positive correlation of CRP levels with positive blood cultures. Increased CRP levels were associated with increased probability of bacterial infection in the blood;
- The use of CRP test together with IMCI guidelines may improve rational use of antimicrobial drugs in settings with limited diagnostic facilities.
